# Exploring the interplay of prolactin and bromocriptine on serum markers in granulomatous lobular mastitis

**DOI:** 10.3389/fimmu.2025.1608875

**Published:** 2025-08-29

**Authors:** Binheng Liu, Wenchao Qu, Yi Feng, Xueqing Wu, Jiamei Feng, Qingqian Gao, Shijun Shao, Jiaye Sun, Hua Wan, Weiping Chen

**Affiliations:** ^1^ Faculty of Chinese Medicine, Macau University of Science and Technology, Taipa, Macau SAR, China; ^2^ Mammary Department, Shuguang Hospital Affiliated to Shanghai University of Traditional Chinese Medicine, Shanghai, China; ^3^ Faculty of Chinese Medicine, Shanghai University of Traditional Chinese Medicine, Shanghai, China

**Keywords:** granulomatous lobular mastitis, prolactin, immune, inflammatory, pathogenesis, ceruloplasmin

## Abstract

**Objective:**

In this study, we retrospectively analyzed the relationship between PRL level and serum inflammatory and immune markers in patients with granulomatous lobular mastitis (GLM) and analyzed the effect of bromocriptine treatment on serum inflammatory and immune markers in patients with GLM. These analyses were conducted to illustrate that PRL is not only an endocrine hormone but also an immune factor, thereby providing evidence that GLM is an autoimmune disease.

**Methods:**

We conducted a retrospective analysis of GLM cases admitted between 2023 and 2024. Clinical features were compared between patients with differential prolactin (PRL) levels using nonparametric tests, with concomitant documentation of prevalent clinical manifestations. Spearman’s rank correlation was employed to assess associations between PRL concentrations and clinical characteristics/serum biomarkers. To evaluate bromocriptine’s therapeutic efficacy, a propensity score-matched (PSM) cohort was established. Longitudinal serological changes were analyzed using nonparametric statistical methods for paired comparisons.

**Results:**

Elevated prolactin levels significantly correlated with lesion size (p<0.05). Patients with abnormal PRL exhibited lower 6-month cure rates compared to those with normal levels (93.1% *vs.* 100%, p=0.02). Baseline-PRL positively associated with neutrophil counts (NE#), Immunoglobulin E (IgE), and ceruloplasmin (CER) (all p<0.05). After treatment, baseline-PRL remained linked to elevated neutrophils, C-reactive protein (CRP), erythrocyte sedimentation rate (ESR), T4 lymphocyte percentage (CD4+ T cells), and IgE, but inversely correlated with lymphocytes (all p<0.05). Propensity-matched analysis (17 bromocriptine-treated *vs*. 13 no-treated) revealed reduced ESR, downregulated major histocompatibility complex class II (MHC-II) expression, and increased interleukin 4 (IL4), interleukin 5 (IL5), and regulatory T cell percentage (Treg%) levels in the treatment group (all p<0.05).

**Conclusions:**

Our findings suggest prolactin may act as an immunomodulatory factor in GLM, potentially influencing T/B-cell immunity and inflammatory cytokine recruitment. Additionally, the observed correlation between prolactin and ceruloplasmin positions ceruloplasmin as a candidate biomarker for GLM, though further validation in independent cohorts is required.

## 1 Introduction

Granulomatous lobular mastitis, a benign chronic inflammatory disorder of mammary glandular tissue (1), demonstrates a prevalence of 3.5% in reproductive-aged populations (2). This condition predominantly manifests 3–5 years postpartum, persisting for 5–15 months (extending to 24 months in refractory cases) (3), with documented recurrence rates spanning 24.8-59% (4–6). While GLM is non-lethal, its severe symptomatology, protracted disease course, and high recurrence rates impose substantial physical and psychological burdens on patients (7). Although the precise etiology and pathogenesis remain undefined, GLM demonstrates strong epidemiological associations with pregnancy and hyperprolactinemia (1). The clinical characteristics of mammary inflammation, erythema nodosum, and arthritis, coupled with its enigmatic pathogenesis, have led to the prevailing hypothesis that GLM is an autoimmune entity. Prolactin, a pituitary-derived polypeptide hormone traditionally associated with lactogenesis and mammary gland development, demonstrates pleiotropic regulatory functions in both physiological and pathological contexts. Emerging evidence reveals PRL’s dual immunoregulatory capacity, functioning both as an endocrine hormone and a cytokine capable of precipitating immune hyperactivation (8–10). Clinically, bromocriptine (a dopamine D2 receptor agonist) serves dual therapeutic roles: as first-line management for hyperprolactinemia and as an immunomodulatory adjunct in GLM through PRL suppression. The demonstrated clinical efficacy of PRL normalization in GLM treatment provides compelling indirect evidence of their pathobiological interconnection.

This study retrospectively analyzed clinical and laboratory data of recent patients to investigate prolactin’s correlations with etiological factors, local symptoms, and serum biomarkers in GLM. We further examined the prognostic implications of baseline PRL levels on postoperative inflammatory/immune responses and evaluated bromocriptine’s therapeutic effects on immunoinflammatory parameters, aiming to investigate the potential association of PRL with these responses and to provide evidence supporting the hypothesis that GLM may be an autoimmune disease.

## 2 Patients and methods

### 2.1 Clinical data

Clinical records were retrospectively collected from patients hospitalized at Shuguang Hospital, Shanghai University of Traditional Chinese Medicine between January 2023 and December 2024.

### 2.2 Diagnostic criteria

All cases were pathologically diagnosed with GLM according to the criteria defined in “Management of GLM: An international multidisciplinary consensus (2021 edition)” (1). As per the consensus:”GLM is characterized histologically as non-caseating granulomatous lesions with epithelioid histiocytes and multinucleated giant cells, located in the center of the lobes. The surrounding tissue is mainly infiltrated by neutrophils, lymphocytes, plasma cells and a small number of eosinophils. The lesions can be multifocal and form micro-abscesses and vary in size.

### 2.3 Inclusion criteria

1. Biological females aged ≥18 years;
2. Histopathological confirmed GLM diagnosis (per institutional protocols);
3. Hospitalized patients receiving standardized care protocols with: Complete clinical documentation (symptom progression, physical findings); Comprehensive laboratory profiles (pre/post-operative inflammatory and immune biomarkers).

### 2.4 Exclusion criteria

1. Patients with incomplete medical records or missing essential laboratory data;
2. Individuals with concurrent severe comorbidities or undergoing therapies that may confound study outcomes;
3. Pregnant and lactating women.

### 2.5 Therapeutic protocol

All enrolled patients underwent protocol-defined surgical interventions coupled with standardized postoperative care. Participants in the bromocriptine cohort received supplemental perioperative administration of bromocriptine mesylate (2.5 mg orally twice daily) as adjuvant therapy. The control group received no adjuvant pharmacological therapy.

### 2.6 Data collection

Baseline clinical characteristics of the enrolled patients were documented, encompassing age, body mass index (BMI), postpartum and lactation periods, trauma and menstrual history, as well as smoking habits. Disease-related symptoms such as fever, leg erythema, arthritis, breast mass size, skin changes, suppuration, medication, and duration were also recorded. Additionally, serum laboratory data were obtained upon admission and after 14 days of treatment, including complete blood count, ESR, CRP levels, lymphocyte subsets, interleukins, and immunoglobulin. Follow-up assessments were conducted at 2-,3- and 6-month post-treatment to monitor the recovery progress of the patients.

### 2.7 Propensity score matching

PSM was performed to balance baseline characteristics between bromocriptine-treated and untreated cohorts, using 1:1 nearest-neighbor matching with a caliper width of 0.2 SD. Covariates included age, BMI, disease duration, lesion size, ulceration number, quadrant involvement, febrile status, erythema severity grading, trauma history, and pretreatment prolactin levels.

### 2.8 Statistical methods

Statistical analyses were performed using SPSS 23.0 and Origin-Pro 2025. Continuous variables with non-normal distribution (assessed by Shapiro-Wilk test) are presented as median (interquartile range) and analyzed using Mann-Whitney U/Kruskal-Wallis tests. Categorical variables are expressed as frequencies (%) with Pearson’s χ² or Fisher’s exact tests for comparisons. Spearman’s rank correlation coefficient was employed for bivariate analyses. Statistical significance was defined as two-tailed p<0.05.

### 2.9 Flowchart

**Figure 1:**
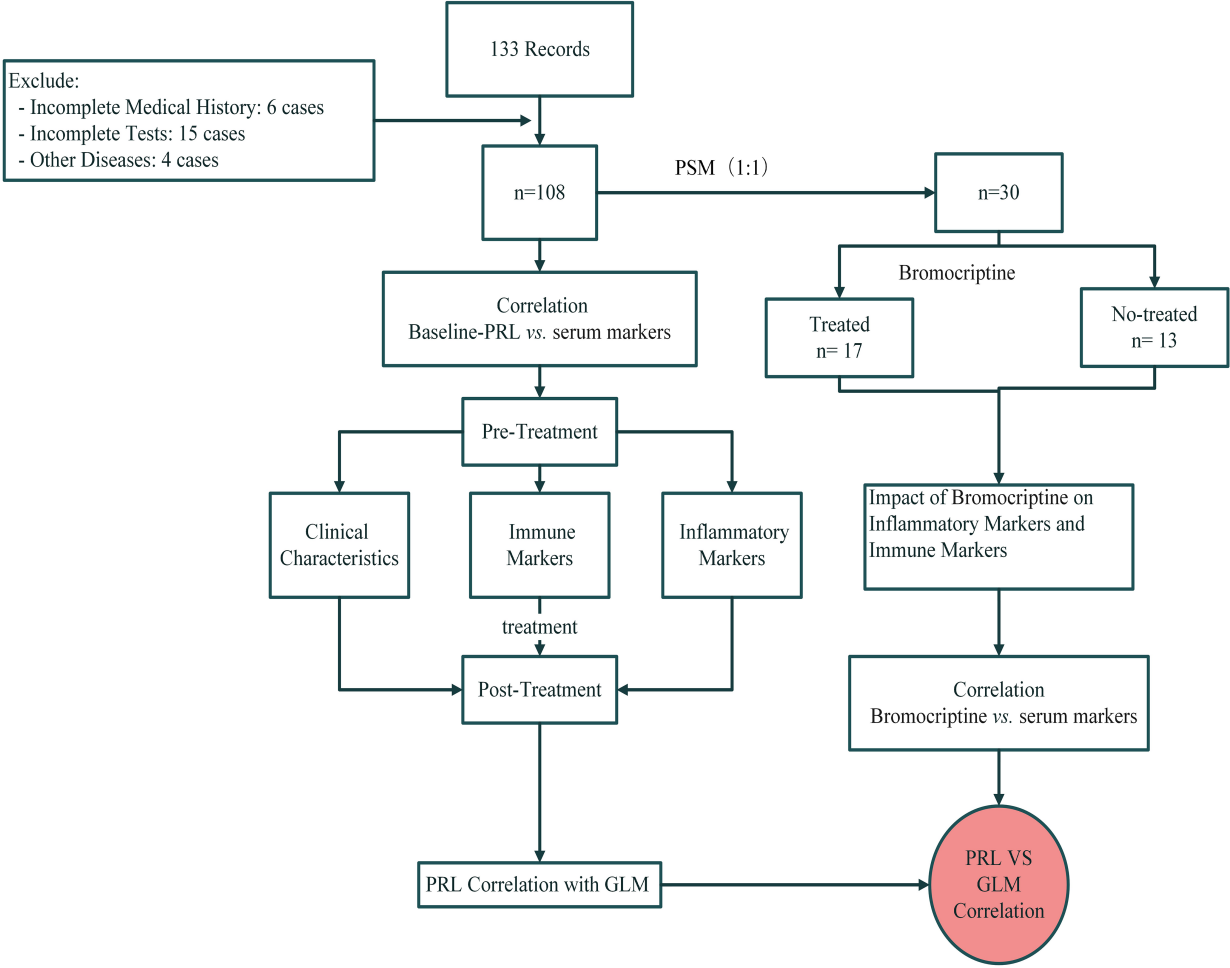
The flowchart of prolactin and bromocriptine on serum markers in Granulomatous Lobular Mastitis.

The flow chart of this study is shown in **Figure 1**.

## 3 Results

### 3.1 Demographic and clinical characteristics

A total of 108 women meeting the inclusion criteria were included in the study, as detailed in **Table 1**. These women had a mean (SD) age of 33.76 (5.28) years and a BMI of 24.44 (3.95) kg/m². Stratified by serum PRL status, both groups had similar median BMI values close to the upper limit of normal (24.00 kg/m²), a median postpartum interval of 4 years, lesion sizes larger than 10 cm, median lactation duration exceeding 6 months, and disease duration surpassing 2 months. Notably, the group with PRL dysregulation exhibited significantly larger breast lesions compared to the other group (median 12 cm *vs*. 10 cm, p=0.05) (**Table 1**).

**Table 1:** Patient characteristics and local breast symptoms.

Variable	PRL, M (IQR)		MannWhitney-U	MannWhitney-z	p
	Normal (n=79)	Abnormal (n=29)			
Demographic characteristics					
Age (year)	34.00 (8.00)	33.00 (7.00)	1101.5	-0.306	0.76
BMI (kg/m^2^)	24.03 (5.67)	24.84 (5.12)	989.5	-1.081	0.28
Postpartum interval (year)	4.50 (4.00)	4.00 (4.25)	877	-0.858	0.39
Lactation duration (month)	11.00 (4.00)	8.00 (9.25)	740	-1.922	0.06
Disease duration (month)	2.00 (2.00)	2.00 (2.00)	1124.5	-0.153	0.88
Local breast symptoms					
Mass size (cm)	10.00 (6.00)	12.00 (6.00)	864.5	-1.956	0.05*
Involved quadrants (n)	3.00 (1.00)	3.00 (0.50)	920	-1.767	0.08
Erythema diameter (n)	4.00 (5.00)	4.00 (3.00)	1068.5	-0.54	0.59
Ulcerative foci (n)	0.00 (1.00)	1.00 (1.00)	1070.5	-0.581	0.56

*p=0.05; n, number of subjects.

**Figure 2:**
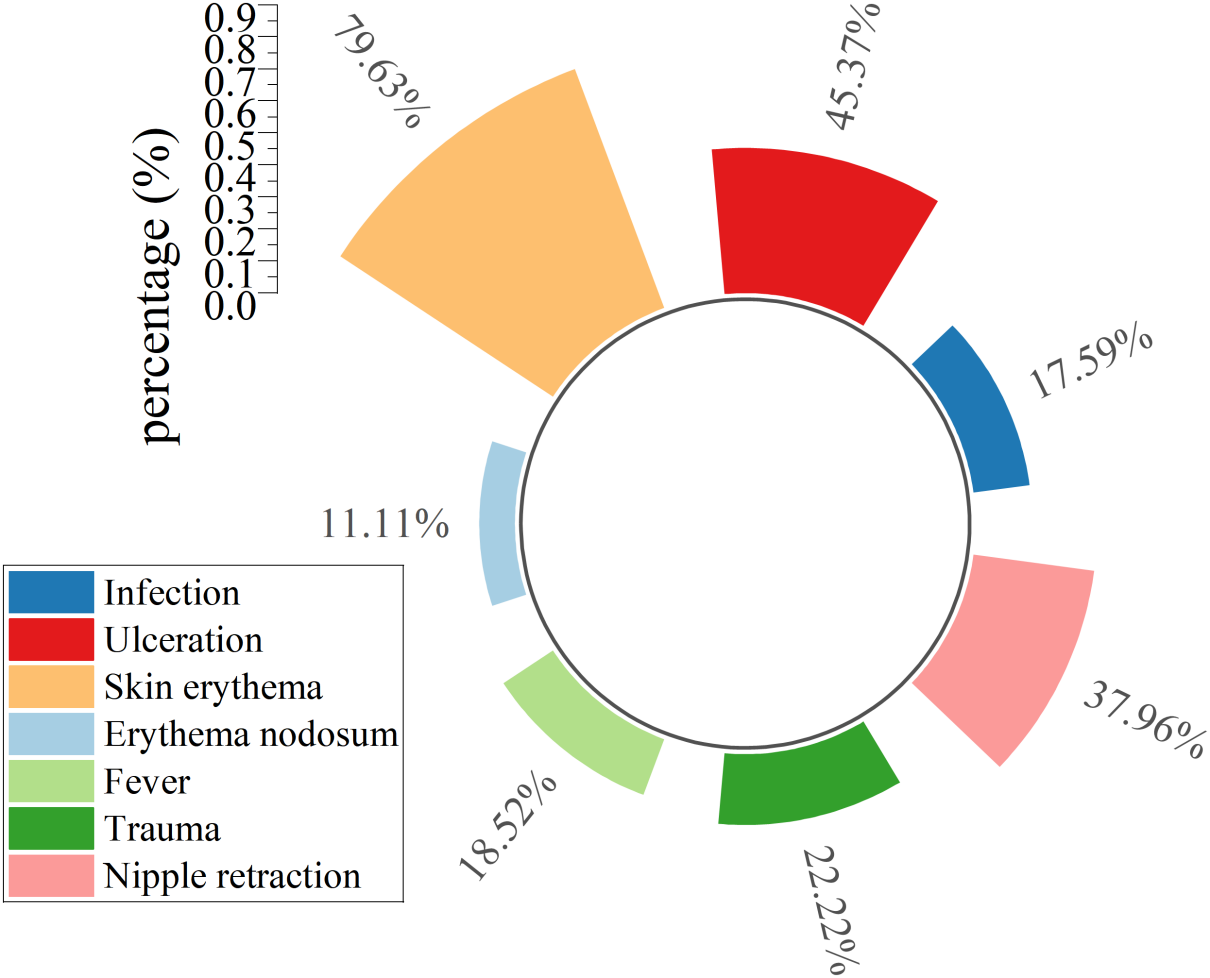
Clinical manifestations.

Local breast manifestations (excluding masses) and systemic symptoms were systematically cataloged in **Figure 2**. Local breast findings including erythema (79.63%), ulceration (45.37%), and nipple retraction (37.96%) demonstrated significantly higher prevalence than systemic manifestations such as fever (18.52%) and erythema nodosum (11.11%). Bacterial infections (17.59%) as the second least frequent complication throughout clinical management, marginally exceeding erythema nodosum (11.11%) (**Figure 2**).

### 3.2 Correlation between baseline-PRL and GLM

#### 3.2.1 Baseline-PRL levels and prognosis

Longitudinal follow-up evaluations were conducted at 2-, 3-, and 6-month post-treatment and systematically examined (**Table 2**). Patients exhibiting prolactin dysregulation showed notably lower rates of remission at the 6-month follow-up in comparison to patients with normal prolactin levels (**Table 2**).

**Table 2:** PRL levels and prognosis.

Follow-up time (Month)	Prognosis (Cured ^b^)	PRL (n, %)		Total	χ2	p
		Normal	Abnormal			
2^a^	Yes	76 (96.20)	27 (93.10)	103 (95.37)	0.46	0.50
	No	3 (3.80)	2 (6.90)	5 (4.63)		
3^a^	Yes	76 (96.20)	27 (93.10)	103 (95.37)	0.46	0.50
	No	3 (3.80)	2 (6.90)	5 (4.63)		
6^a^	Yes	79 (100.00)	27 (93.10)	106 (98.15)	5.55	0.02*
	No	0 (0.00)	2 (6.90)	2 (1.85)		

*p<0.05.

a Months Post-Treatment.

b Clinical cure refers to the disappearance of clinical symptoms and the complete healing of wounds or lesions.

#### 3.2.2 Correlations between baseline-PRL levels and inflammatory markers

**Figure 3:**
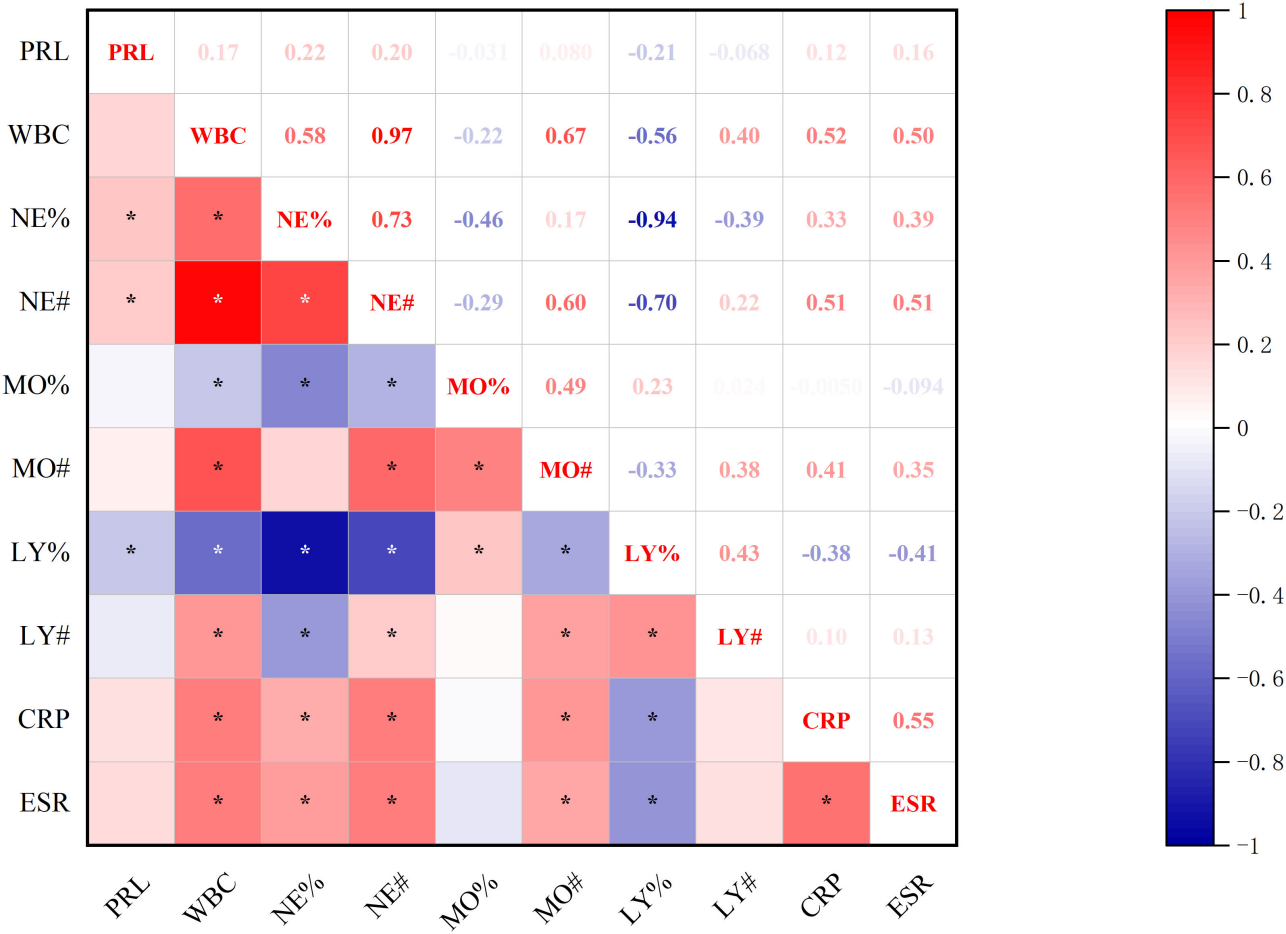
Correlations between baseline-PRL Levels and inflammatory markers. *p<0.05; PRL, prolactin blood level; WBC, white blood cell count; NE%, neutrophil percentage; NE#, neutrophil count; MO%, monocyte percentage; MO#, monocyte count; LY%, lymphocyte percentage; LY#, lymphocyte count; CRP, C-reactive protein; ESR, erythrocyte sedimentation rate.

Significant correlations emerged between baseline prolactin levels and inflammatory indices (**Figure 3**): positive associations with neutrophilic markers (both NE% and NE#) contrasted with negative correlation to lymphocyte percentage (LY%) (all p<0.05, Spearman’s test).

#### 3.2.3 Correlations between baseline-PRL levels and immunoglobulins

**Figure 4:**
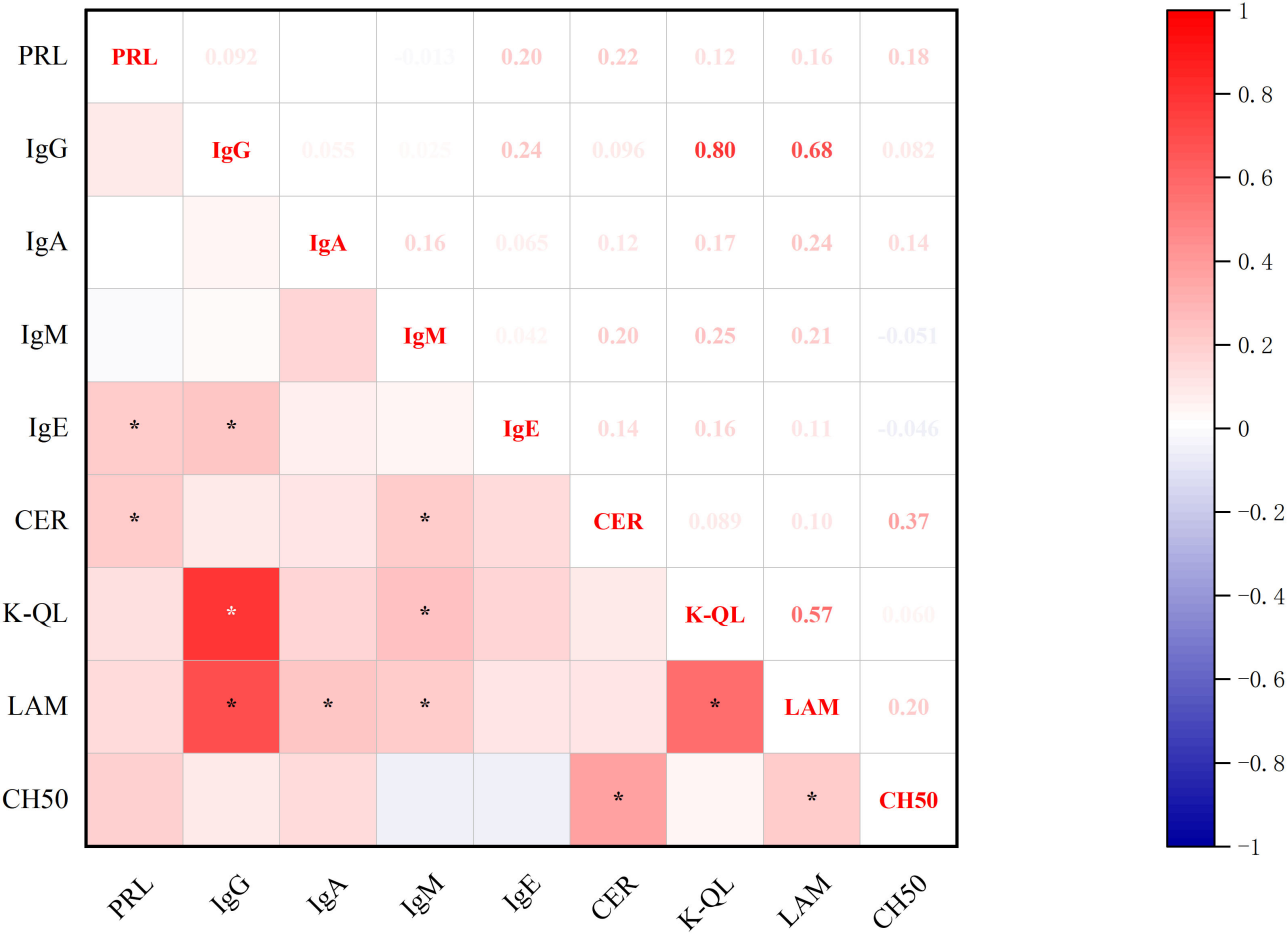
Correlations between PRL Levels and immunoglobulin. *p<0.05; PRL, prolactin blood level; IgG, immunoglobulin G; IgA, immunoglobulin A; IgM, immunoglobulin M; IgE, immunoglobulin E; CER, ceruloplasmin; K-QL, Kappa light chain; LAM, Lymphocyte Activation Markers; CH50, Complement system components.

Significant correlations emerged between baseline prolactin levels and immunological markers (**Figure 4**): positive associations were observed with IgE and CER. Furthermore, CER demonstrated additional correlations with IgM and complement system components (CH50), while IgE showed a positive association with IgG. PRL was not significantly associated with Interleukin and lymphocyte subsets (all p<0.05, Spearman’s test).

#### 3.2.4 Correlations between baseline-PRL levels and clinical features

**Figure 5:**
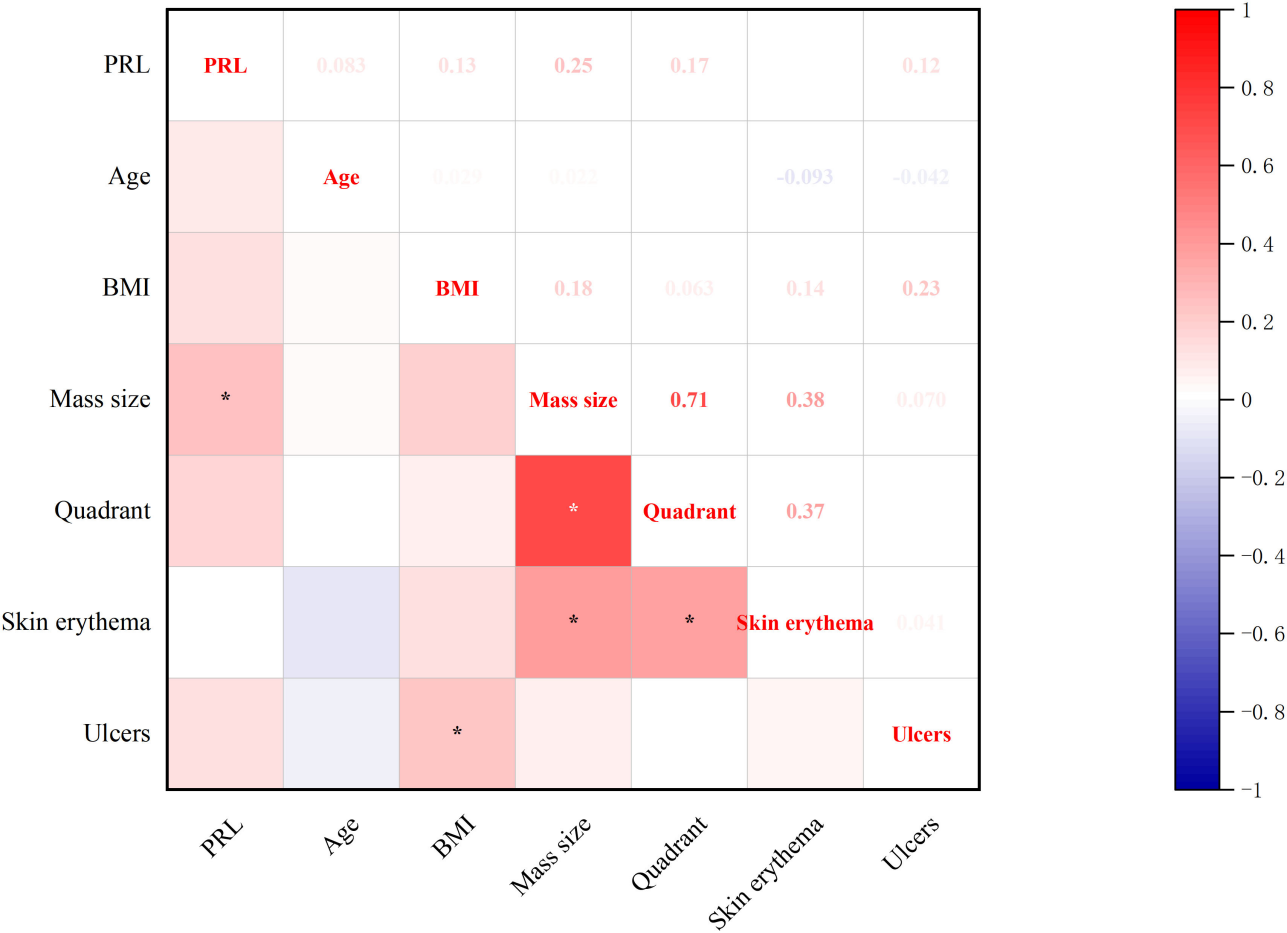
Correlations between baseline-PRL Levels and clinical features. *p<0.05.

Significant correlations emerged between baseline prolactin levels and clinical features (**Figure 5**): direct associations were observed with breast mass size. Concurrently, BMI showed positive correlations with ulcers which present suppurative breast lesion development (all p<0.05, Spearman’s test).

### 3.3 Correlations between baseline-PRL and GLM serum markers after treatment

#### 3.3.1 Correlations between baseline-PRL and post-treatment inflammatory makers

**Figure 6:**
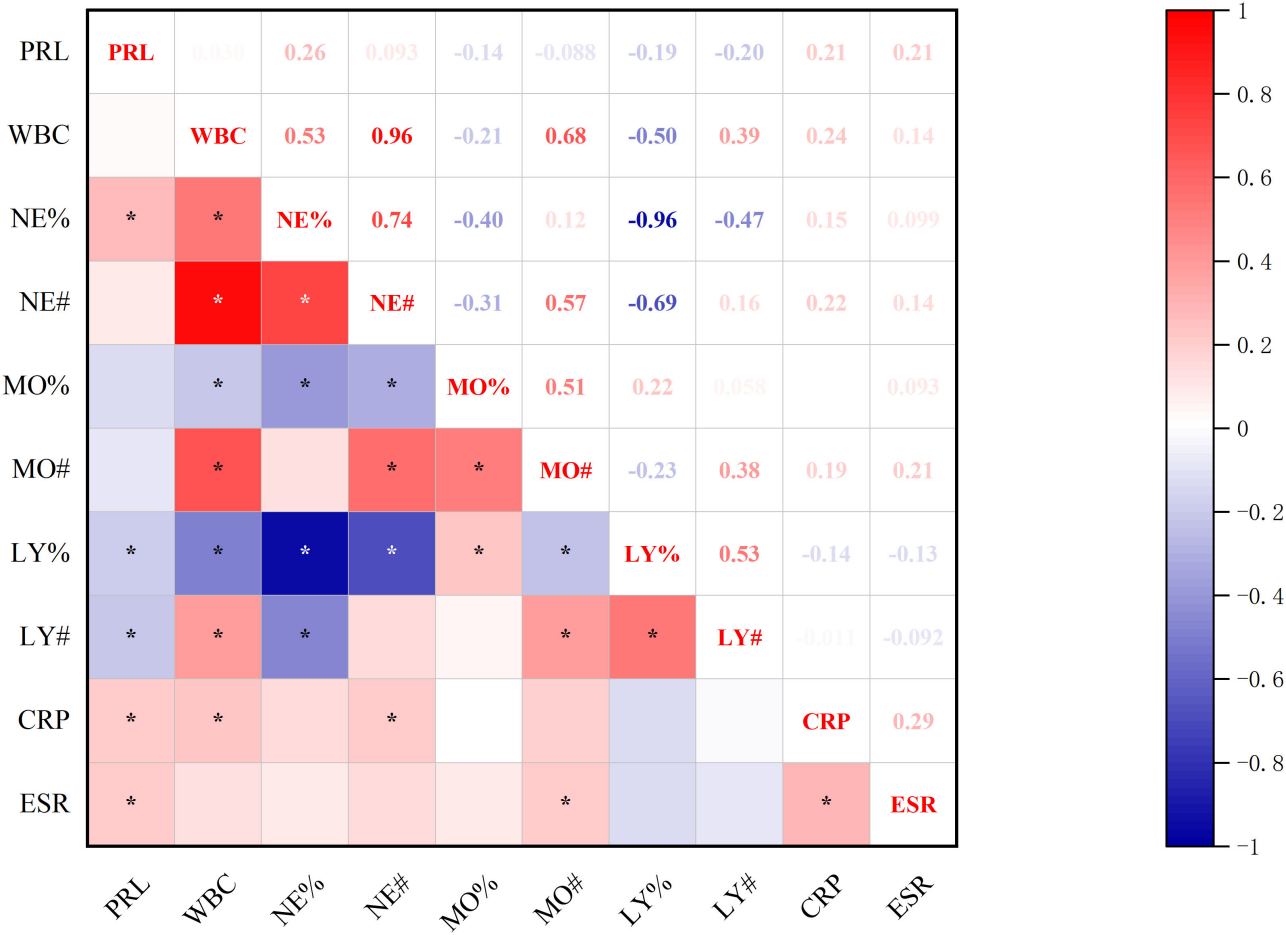
Correlation between baseline-PRL and post-treatment inflammatory makers. *p<0.05; PRL, prolactin blood level; WBC, white blood cell count; NE%, neutrophil percentage; NE#, neutrophil count; MO%, monocyte percentage; MO#, monocyte count; LY%, lymphocyte percentage; LY#, lymphocyte count; CRP, C-reactive protein; ESR, erythrocyte sedimentation rate.

Significant correlations were observed between baseline-PRL levels and post-treatment inflammatory markers, including complete blood count parameters, CRP, ESR (**Figure 6**). PRL demonstrated positive associations with NE%, CRP, and ESR, while showing a negative correlation with lymphocyte (both LY# and LY%) (all p<0.05, Spearman’s test).

#### 3.3.2 Correlations between baseline-PRL and post-treatment immune molecules

**Figure 7:**
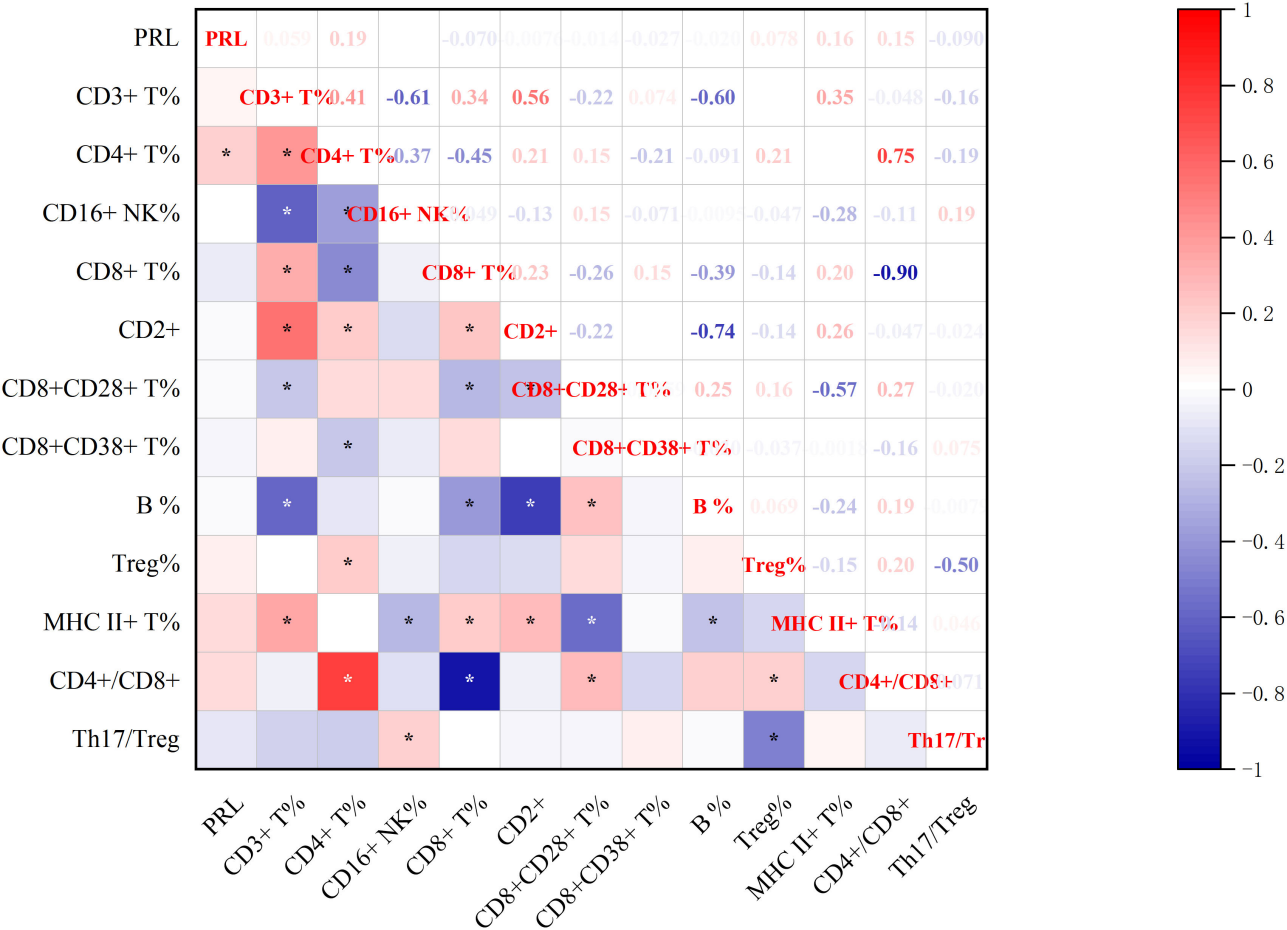
Correlation between baseline-PRL and post-treatment Lymphocyte Subsets.*p<0.05; CD3+ T%, total T lymphocyte percentage; CD4+ T%, T4 lymphocyte percentage; CD16+ NK%, NK lymphocyte percentage ; CD8+ T%, T8 lymphocyte percentage; CD2+, leukocyte function-associated antigen; CD8+CD28+ T%, CD28+ T8 lymphocytes ; CD8+CD38+ T%,CD38+ T8 lymphocytes ; CD19+ B%, B lymphocyte percentage; Treg%, regulatory T cell percentage; MHC II+ T%, MHC-II T cells; CD4+/CD8+,CD4/CD8 T4/T8 lymphocyte ratio; Th17/Treg, helper-T 17/ regulatory T cell radio.

**Figure 8:**
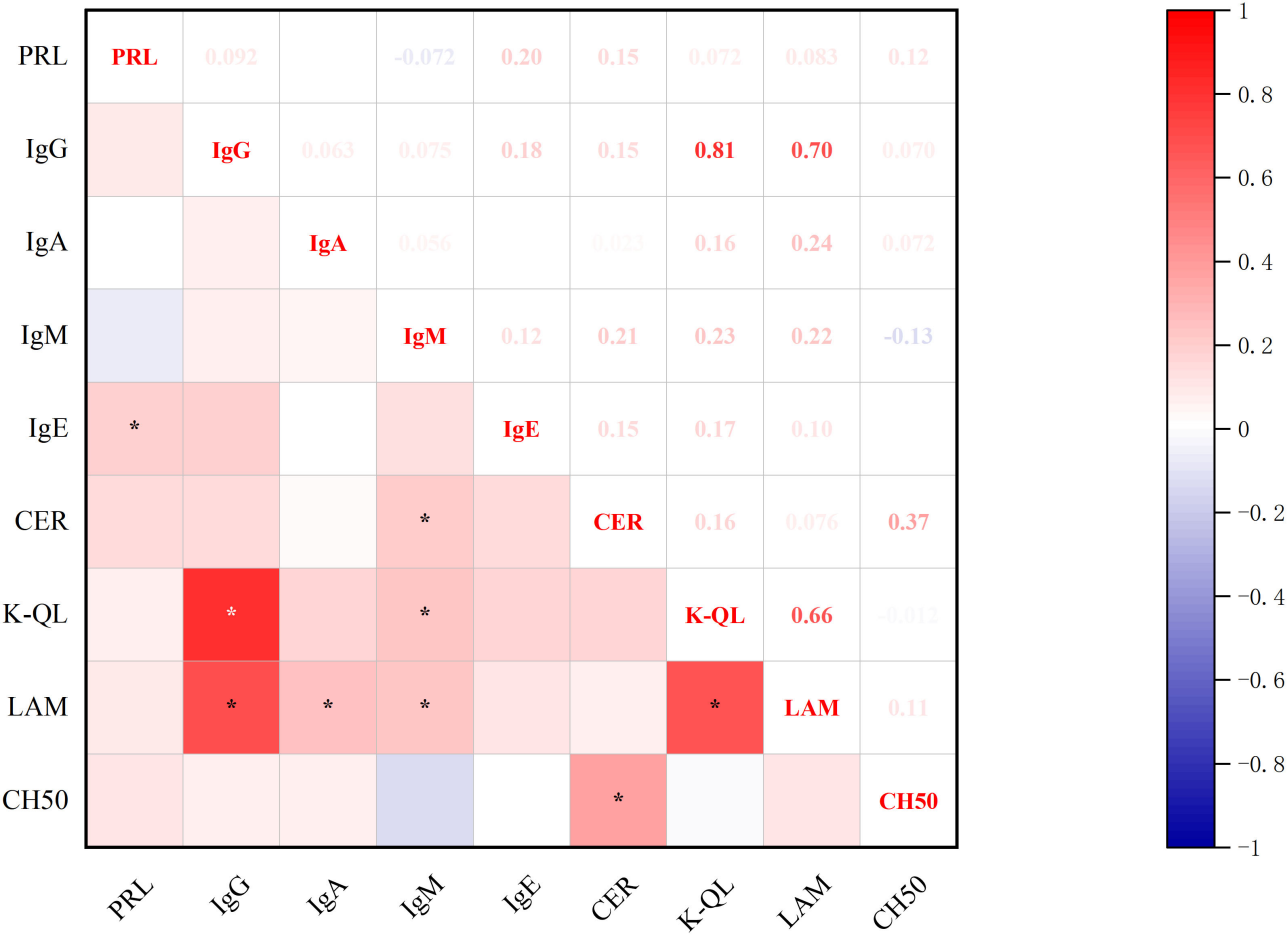
Correlations between baseline-PRL and post-treatment immunoglobulins. *p<0.05; PRL, prolactin blood level; IgG, immunoglobulin G; IgA, immunoglobulin A; IgM, immunoglobulin M; IgE, immunoglobulin E; CER, ceruloplasmin; K-QL, kappa light chain; LAM, lymphocyte activation markers; CH50, complement system components.

Significant correlations emerged between baseline-PRL levels and post-treatment immune profiles (**Figures 7**, **8**). PRL demonstrated positive associations with CD4+ T% and IgE. Furthermore, CD4+ T% showed positive correlations with CD3+ T%, CD2+, Treg%, and CD4+/CD8+, while negative correlations with CD16+ NK%, CD8+ T%, and CD8+CD38+ (all p<0.05, Spearman’s test).

### 3.4 Propensity-matched analysis of the effect of bromocriptine on GLM

To elucidate the PRL-GLM interaction, we conducted PSM using clinical features in the study cohort. Patients were stratified into bromocriptine-treated (n=17) and untreated control (n=13) groups based on therapeutic regimen (**Table 3**).

**Table 3:** Baseline clinical features before and after PSM.

Variable	Before-matching Bromocriptine		p	After-matching Bromocriptine		p
	No-treated (n=91)	Treated (n=17)		No-treated (n=13)	Treated (n=17)	
Age (year, mean ± SD)	33.54 ± 5.29	34.94 ± 5.24	0.32	35.62 ± 4.74	34.94 ± 5.24	0.72
BMI (kg/m^2^, mean ± SD)	24.38 ± 4.14	24.79 ± 2.76	0.70	23.69 ± 3.82	24.79 ± 2.76	0.37
Disease duration (month, mean ± SD)	2.34 ± 1.97	2.06 ± 1.34	0.47	2.23 ± 1.24	2.06 ± 1.34	0.72
Mass size (cm, mean ± SD)	10.46 ± 4.13	12.12 ± 6.35	0.31	12.15 ± 4.16	12.12 ± 6.35	0.99
Ulcerative foci (n, mean ± SD)	0.65 ± 0.99	0.65 ± 0.70	1.00	0.54 ± 0.52	0.65 ± 0.70	0.64
Involved quadrants (n, mean ± SD)	2.85 ± 0.68	2.94 ± 0.66	0.60	3.00 ± 0.58	2.94 ± 0.66	0.80
Fever						
No (n,%)	73 (80.22)	15 (88.24)	0.39	11 (84.62)	15 (88.24)	0.79
Yes (n,%)	18 (19.78)	2 (11.76)		2 (15.38)	2 (11.76)	
Erythema						
No (n,%)	80 (87.91)	16 (94.12)	0.37	12 (92.31)	16 (94.12)	0.85
Yes (n,%)	11 (12.09)	1 (5.88)		1 (7.69)	1 (5.88)	
Trauma						
No (n,%)	71 (78.02)	13 (76.47)	0.89	10 (76.92)	13 (76.47)	0.98
Yes (n,%)	20 (21.98)	4 (23.53)		3 (23.08)	4 (23.53)	
PRL						
Normal (n,%)	71 (78.02)	8 (47.06)	0.02*	7 (53.85)	8 (47.06)	0.72
Abnormal (n,%)	20 (21.98)	9 (52.94)		6 (46.15)	9 (52.94)	

*p<0.05.

#### 3.4.1 Effect of bromocriptine on inflammatory markers

In the propensity-matched cohort, the bromocriptine-treated group demonstrated significantly lower prolactin levels and ESR compared to untreated controls (**Table 4**).

**Table 4:** Effect of bromocriptine on inflammatory markers.

Inflammatory markers	Bromocriptine M (IQR)		MannWhitney-U	MannWhitney-z	p
	Treated (n=17)	No-treated (n=13)			
WBC (10^9^/L)	6.47 (2.84)	5.99 (3.31)	110.00	-0.02	0.98
NE%	67.20 (8.50)	65.80 (12.65)	98.00	-0.52	0.60
NE# (10^9^/L)	4.35 (2.72)	3.66 (3.02)	109.00	-0.06	0.95
MO%	6.20 (2.05)	6.80 (2.00)	73.00	-1.57	0.12
MO# (10^9^/L)	0.40 (0.13)	0.45 (0.20)	75.50	-1.47	0.14
LY%	22.60 (8.85)	24.50 (11.65)	104.00	-0.27	0.79
LY# (10^9^/L)	1.49 (0.66)	1.30 (0.61)	87.00	-0.98	0.33
CRP (mg/L)	1.17 (6.21)	0.50 (3.68)	99.00	-0.51	0.61
ESR (mm/h)	34.00 (27.00)	56.00 (51.00)	59.00	-2.16	0.03*

*p<0.05

WBC, white blood cell count; NE%, neutrophil percentage; NE, neutrophil count; MO%, monocyte percentage; MO#, monocyte count; LY%, lymphocyte percentage; LY#, lymphocyte count; CRP, C-reactive protein; ESR, erythrocyte sedimentation rate.

#### 3.4.2 Effect of bromocriptine on immune molecules

In the propensity-matched cohort, the bromocriptine-treated group demonstrated significantly elevated Treg%, IL4, and IL5 compared to untreated controls. Conversely, MHC-II+T cells expression was markedly reduced in the treatment group (**Tables 5**, **6**).

**Table 5:** Effect of bromocriptine on immune molecules.

Immune molecules	Bromocriptine M (IQR)		MannWhitney-U	MannWhitney-z	p
	Treated (n=17)	No-treated (n=13)			
CD3+ T%	80.97 (11.29)	80.63 (8.20)	96.00	-0.61	0.54
CD4+ T%	45.25 (15.17)	43.74 (9.50)	90.50	-0.84	0.40
CD16+ NK%	6.92 (5.10)	6.20 (6.77)	82.00	-1.19	0.23
CD8+ T%	28.22 (9.93)	26.55 (11.28)	110.00	-0.02	0.98
CD2+	84.85 (6.81)	84.05 (7.66)	95.50	-0.63	0.53
CD8+CD28%	67.10 (17.05)	59.50 (19.13)	80.00	-1.28	0.20
CD8+CD38%	37.77 (22.90)	41.38 (13.79)	95.00	-0.65	0.52
CD19+ B%	9.35 (5.43)	10.88 (4.14)	91.00	-0.82	0.41
Treg%	4.62 (3.32)	3.30 (2.65)	59.00	-2.16	0.03*
MHC II+ T	17.70 (8.29)	24.94 (14.97)	55.00	-2.32	0.02*
CD4+/CD8+	1.60 (0.85)	1.60 (0.90)	106.50	-0.17	0.87
Th17/Treg	0.62 (1.88)	1.00 (1.90)	84.00	-1.11	0.27

*p<0.05.

CD3+T%, total T lymphocyte percentage; CD4+ T%, T4 lymphocyte percentage; CD16+ NK%, NK lymphocyte percentage; CD8+ T%, T8 lymphocyte percentage; CD2+, leukocyte function-associated antigen; CD8+CD28+ T%, CD28+ T8 lymphocytes; CD8+CD38+ T%, CD38+ T8 lymphocytes; CD19+ B%, B lymphocyte percentage; Treg%, regulatory T cell percentage; MHC II+ T%, MHC-II T cells; CD4+/CD8+, CD4/CD8 radio; T4/T8 lymphocyte ratio; Th17/Treg, helper-T 17/regulatory T cell radio.

**Table 6:** Effect of bromocriptine on interleukin.

Interleukin	Bromocriptine M (IQR)		MannWhitney-U	MannWhitney-z	p
	Treated (n=17)	No-treated (n=13)			
IL-1β (pg/ml)	4.33 (5.22)	4.33 (21.08)	110.5	0	1
IL2 (pg/ml)	1.56 (2.73)	1.44 (2.14)	83.00	-1.16	0.25
IL4 (pg/ml)	2.00 (0.74)	1.20 (0.89)	62.50	-2.06	0.04*
IL5 (pg/ml)	2.55 (0.89)	1.90 (0.96)	56.50	-2.26	0.02*
IL6 (pg/ml)	2.92 (3.43)	2.31 (2.25)	93.50	-0.71	0.48
IL8 (pg/ml)	3.35 (3.16)	2.49 (1.48)	84.50	-1.10	0.27
IL10 (pg/ml)	1.25 (0.55)	1.38 (0.71)	86.50	-1.01	0.31
IL12 (pg/ml)	1.47 (0.45)	1.27 (0.55)	82.50	-1.18	0.24
IL17 (pg/ml)	2.57 (6.44)	2.73 (4.31)	98.50	-0.50	0.61
IFN-γ (pg/ml)	2.53 (7.43)	2.39 (1.54)	65.00	-1.92	0.05
IFN-α (pg/ml)	1.95 (2.88)	1.62 (1.68)	85.50	-1.05	0.29
TNF-α (pg/ml)	2.19 (1.75)	2.21 (0.15)	100.00	-0.45	0.65

*p<0.05; IL, interleukin; IFN-γ, interferon-gamma; IFN-α, interferon-alpha; TNF-α, tumor necrosis factor-alpha.

## 4 Discussion

### 4.1 GLM: a persistent therapeutic conundrum

Granulomatous lobular mastitis, a benign chronic inflammatory breast condition predominantly affecting women of childbearing age (median 33 years, typically 4 years postpartum), demonstrates prolonged clinical courses (≤2 years) and high recurrence rates (24.8-59%) (1, 3–6). Our surgical cohort exhibited severe disease manifestations: median lesion size 10 cm, multi-quadrant involvement (≥2 quadrants), and suppuration (up to 6 episodes). Localized breast symptoms significantly outweighed systemic manifestations, with bacterial infections implicated in only 17.59% of cases, substantially limiting antibiotic efficacy. These findings underscore the dual burden of GLM - physically debilitating presentations compounded by profound psychosocial distress (7). The urgent need persists for pathogenesis elucidation, diagnostic biomarker development, and targeted therapeutic strategies to mitigate diagnostic delays and optimize clinical management.

### 4.2 PRL dysregulation in GLM progression

Amidst etiological controversies, pregnancy and hyperprolactinemia emerge as the most substantiated GLM correlates (1, 11, 12), demonstrating stronger pathogenic relevance than menstrual irregularities, nipple deformity or obesity (13–17). Emerging evidence consistently implicates prolactin dysregulation in GLM pathogenesis and progression (1). Patients with GLM demonstrate significantly elevated PRL levels compared to healthy controls (18), with lactation-associated hyperprolactinemia identified as a primary risk factor through its promotion of ductal secretion abnormalities (17). Notably, intracranial lesion-induced PRL elevation may directly contribute to GLM development (19), underscoring the importance of routine PRL screening in clinical evaluation. Longitudinal observations further suggest that dynamic changes in PRL levels before and after treatment correlate with recurrence risk (20), highlighting the prognostic significance of PRL monitoring. These collective findings establish PRL modulation as a critical component in comprehensive GLM management strategies (21). Our study shows that PRL abnormalities may affect cure rates 6 months after treatment. Meanwhile, our findings demonstrate that patients with PRL dysregulation present significantly larger breast lesions, with a positive correlation observed between PRL levels and lesion dimensions, reinforcing the pathophysiological link between hyperprolactinemia and disease severity. Importantly, lesion size emerged as a key prognostic indicator: larger lesions correlated with intensified pain perception, broader anatomical involvement, and prolonged recovery duration, consistent with established clinical patterns. Our study provides a foundation for further investigation into the association between PRL and disease severity.

### 4.3 Correlation between PRL and inflammatory markers

Histopathological evaluation reveals GLM is characterized by dense inflammatory cell infiltration surrounding mammary lobules, predominantly comprising neutrophils, lymphocytes, and plasma cells (22). While neutrophils initiate acute inflammatory responses, their excessive accumulation disrupts resolution pathways, perpetuating tissue damage. This persistent inflammatory milieu, potentially modulated by PRL dysregulation, demonstrates dynamic cytokine fluctuations that may fuel disease chronicity (23, 24). It has been found that PRL promotes dendritic cell maturation, enhances Th1-mediated immunity (Th1 immunity), and affects neutrophil function by increasing pro-inflammatory factors (25). Our findings reveal intensified neutrophil recruitment in patients with PRL abnormalities, aligning with GLM’s characteristic histopathological patterns (22). Notably, sustained neutrophilic involvement post-surgery, coupled with positive correlations between PRL levels and inflammatory markers (CRP and ESR), underscores PRL’s role in amplifying inflammatory intensity. Meantime, the consistent inverse relationship between PRL concentrations and lymphocyte proportions across treatment phases suggests PRL is associated with immune imbalance.

To our knowledge, our study is the first to report a positive correlation between prolactin and ceruloplasmin in GLM. As a multicopper oxidase, CER serves as a biomarker of systemic inflammation, oxidative stress, and copper/iron metabolism (26). The association between CER and oxidative stress is mechanistically linked to IL-6 induction, particularly in lipopolysaccharide (LPS)-driven pathogenesis (27, 28). Prior evidence supports CER’s involvement in autoimmune disorders such as rheumatoid arthritis and lupus erythematosus, providing a pathogenic analogy for GLM’s potential autoimmune etiology (29, 30). Notably, serum CER reflects systemic copper/iron metabolism, and recent studies have established a link between GLM and iron dysregulation: bacterial LPS underlies GLM pathogenesis via iron depletion, mammary cell death, and subsequent IL-6 up-regulation (27, 28). Furthermore, CER is implicated in immune processes, with evidence suggesting its association with immune dysregulation. In studies exploring CER as a prognostic biomarker in breast cancer, CER levels were correlated with T-cell activity and macrophage polarization, demonstrating positive associations with M0/M1 infiltration and negative correlations with M2 macrophages (27, 28). Our findings demonstrate a correlation between CER and PRL, which may further underscore the association of CER with immune dysregulation while positioning CER as a candidate disease-specific diagnostic biomarker for GLM.

### 4.4 Correlation between PRL and T-cell Immunity

Lymphocyte subsets encompass functionally distinct T cells, B cells, and NK cells in peripheral blood, defined by unique surface markers. These populations collaboratively regulate immune defense, homeostasis, and surveillance through specialized roles in adaptive and innate immunity. Prolactin can affect the expression of T-bet gene by affecting CD4+ T cells, and activated T-bet can further enhance the expression of CD4+ T cells. CD4+ T cells have been associated with GLM severity and exhibit dynamic changes during therapy (16, 31, 32). Our study revealed a positive correlation between baseline-PRL levels and post-treatment CD4+ T cells, suggesting that PRL fluctuations may modulate CD4+ T cell dynamics and trigger downstream immune cascades.

CD4+ T cells differentiate into functionally distinct subsets (Th1, Th2, Th17, Treg) under specific stimuli. Emerging evidence highlights Th17 cells as key players in granulomatous pathogenesis, with Th17/Treg imbalance being mechanistically linked to autoimmune disorders and directly associated with granuloma formation in GLM (33–35). Treg cells counterbalance inflammatory responses through TGF-β and IL-10 secretion (10, 36). Intriguingly, while baseline-PRL showed no direct correlation with Treg%, its positive association with CD4+ T%, which correlated positively with Treg%, suggests that PRL may indirectly modulate Treg activity via CD4+ T cells intermediaries. Our PSM analysis further demonstrated elevated Treg% post-treatment, indicating PRL suppression enhances Treg-mediated immunoregulation. These findings collectively suggest PRL may constrain Treg activation, thereby exacerbating GLM-related immune dysregulation.

Additionally as a pivotal mediator of inflammatory cell-T cell communication, MHC-II interacts with surface receptors on antigen-presenting cells including dendritic cells, macrophages, and B lymphocytes (37, 38).In our outcomes, bromocriptine induced PRL reduction coincided with diminished MHC-II expression in treated patients, suggesting a potential interplay between PRL signaling and antigen presentation pathways. This observation, coupled with parallel declines in CRP and ESR, implies PRL-mediated immunomodulation may involve MHC-II-related mechanisms.

### 4.5 Correlation between PRL and B-cell immunity

The immune pathogenesis of GLM involves both innate and adaptive immunity (10, 18, 39, 40). B cells differentiate into plasma cells that secrete immunoglobulins such as IgG and IgE. Notably, prolactin receptors (PRLR) are expressed on B cells at various developmental stages, and dysregulated PRL levels can alter B-cell proliferation and immune function (41). Our findings demonstrate a persistent positive correlation between PRL and IgE levels, even postoperatively. The characteristic histopathological feature of plasma cell infiltration in GLM further confirms adaptive immune activation. Given that plasma cell abundance reflects tissue inflammation severity, consistent with the observed PRL inflammation marker correlations. We propose that PRL may contribute to GLM pathogenesis by modulating B-cell activity.

In our PSM cohort, PRL suppression therapy led to significant increases in IL-4 and IL-5 levels, indicating Th2-mediated immunity (Th2 immune) activation. This finding aligns with prior studies demonstrating that PRL modulates Th2 immune responses to influence disease progression (42), thereby supporting both the “PRL-Th2 drives GLM pathogenesis” hypothesis and providing new evidence for the “Th1/Th2 imbalance contributes to GLM development” theory (32). Notably, IL-4 plays a unique role in tissue repair: it promotes healing by inducing macrophage M2 polarization and stimulating the secretion of anti-inflammatory factors such as IL-10 (38). The observed elevation of IL-4 post-bromocriptine treatment in our study likely reflects dynamic reprogramming of macrophage polarization states, which strongly aligns with the mechanistic model that “macrophage phenotype switching regulates GLM progression and resolution.

### 4.6 PRL: a dual-function hormone and immune cytokine

Prolactin, a multifunctional molecule with a molecular weight of approximately 23 kDa, is secreted predominantly by the pituitary gland but can also be locally synthesized in extra pituitary tissues, including peripheral immune cells and mammary glands (8). The PRLR is widely expressed on the surface of diverse immune cells, such as lymphocytes (particularly natural killer cell subsets), macrophages, monocytes, granulocytes, and thymic epithelial cells, providing a structural basis for PRL’s direct regulation of immune activities (9, 43, 44). By binding to PRLR, PRL activates the Janus kinase 2/signal transducer and activator of transcription 5 (JAK2/STAT5) signaling pathway, which not only enhances Th1/Th17 responses and suppresses Treg function but also modulates NK cell cytotoxicity and macrophage polarization (8, 10, 43–47). Notably, patients with GLM often exhibit systemic manifestations such as arthritis and lower limb erythema, clinical features that closely resemble those of classic autoimmune diseases (10, 44). This observation suggests that PRL may serve as a key regulatory factor linking localized inflammation in GLM to systemic immune dysregulation. The broad distribution of PRLR underscores PRL’s unique role as an immunoendocrine bridge molecule, transcending its traditional perception as a lactation associated hormone (8, 43, 44).

Our study demonstrates that PRL directly modulates inflammatory and immune responses in GLM, as evidenced by its significant positive correlations with neutrophil activity and IgE proliferation (8, 44). In the PSM cohort, PRL suppression therapy reduced MHC-II expression while enhancing Treg, IL4 and IL5 differentiation, indicating PRL’s regulatory role in immune signaling and tolerance (10, 42). These findings provide novel insights into the persistent inflammatory microenvironment of GLM.

## 5 Conclusion

The etiology and pathogenesis of GLM remain incompletely understood, yet our findings strongly support the emerging autoimmune hypothesis by repositioning prolactin as a multifunctional immunomodulator rather than a conventional endocrine hormone. Clinically, PRL levels correlated with lesion size, neutrophil infiltration, CER, post-treatment CD4+ T cells and IgE levels, suggesting its dual role in both innate and adaptive immune dysregulation. Through PSM analysis, bromocriptine-induced PRL suppression was associated with reduced inflammatory markers (ESR and MHC-II) and enhanced Treg/Th2 responses (elevated Tregs, IL-4, IL-5). These observations reveal PRL’s central role in orchestrating immune homeostasis, where its dysregulation may perpetuate GLM pathology through intertwined inflammatory and immunoregulatory pathways. Our work not only provides clinical validation for PRL-driven autoimmunity in GLM but also highlights its potential as a therapeutic target for rebalancing immune tolerance. Additionally, we first report CER dysregulation in GLM, which provides a new perspective for GLM research.

### 5.1 Limitations

This study has several limitations. First, while our results demonstrate correlations between PRL and inflammatory/immune factors, these relationships require validation through basic research to elucidate the underlying mechanisms and molecular targets. Second, the retrospective design and hospital-based case selection may limit the robustness of certain findings—for instance, the association between lactation duration and PRL levels showed borderline significance (P=0.06). Although PSM mitigated confounding effects, its efficacy was constrained by the original cohort size, underscoring the need for larger-scale studies with prospective designs to enhance scientific validity. Additionally, the 2-week post-treatment observation window likely captured only a transient snapshot of immunological changes rather than the complete trajectory of GLM resolution. These limitations highlight the necessity of prospective multicenter studies with extended follow-up periods and standardized protocols to fully characterize PRL’s mechanistic role in GLM pathogenesis.

### 5.2 Future perspectives

Despite these limitations, our findings provide a crucial foundation for advancing GLM research and clinical management. Building on this work, future studies should prioritize expanding sample sizes through multi-center collaborations to enhance statistical validity. Integrating the emerging concept of GLM staging systems could enable mechanistic exploration across disease phases, offering insights into stage-specific pathophysiology. Prospective longitudinal designs are needed to clarify causal relationships between PRL dynamics and GLM progression. Furthermore, experimental models (*e.g.*, immune cell co-cultures and GLM animal models) could unravel PRL’s direct immunomodulatory effects.

## Data Availability

The raw data supporting the conclusions of this article will be made available by the authors, without undue reservation.
